# How effective are mobile apps in managing people with type 2 diabetes mellitus? A systematic literature review protocol

**DOI:** 10.1371/journal.pone.0301523

**Published:** 2024-04-25

**Authors:** Carla Taramasco, Carla Rimassa, María Elena Lagos Garrido, Rosa L. Figueroa

**Affiliations:** 1 Instituto de Tecnologías para la Innovación en Salud y Bienestar (ITiSB), Faculty of Engineering, Universidad Andrés Bello, Viña del Mar, Chile; 2 Millennium Nucleus on Sociomedicine, Santiago, Chile; 3 Escuela de Fonoaudiología, Facultad de Medicina, Campus San Felipe, Universidad de Valparaíso, San Felipe, Chile; 4 Department of Fundamentals and Public Health, Faculty of Nursing, Universidad de Concepción, Bío Bío, Chile; 5 Department of Electrical Engineering, Faculty of Engineering, Universidad de Concepción, Bío Bío, Chile; FMUP: Universidade do Porto Faculdade de Medicina, PORTUGAL

## Abstract

**Introduction:**

The rise of new technologies in the field of health is yielding promising results. In certain chronic conditions such as type 2 diabetes mellitus, which ranks among the top five causes of global mortality, it could be useful in supporting patient management.

**Materials and methods:**

A systematic review will be conducted on scientific publications from the last 5 years (January 2019 to October 2023) to describe the effect of mobile app usage on glycated hemoglobin for the management of adult patients with type 2 diabetes mellitus who participated in randomized controlled clinical trials. The search will be carried out in the databases of MEDLINE (Ovid), Embase (Ovid), CINAHL (EBSCOhost), CENTRAL, WoS, Scopus, Epistemonikos, and LILACS. The search strategy will be constructed using both controlled and natural language. Additionally, the Cochrane filter will be applied to identify randomized controlled trials. The review will include scientific articles reporting studies that present results from randomized controlled trials, with texts in Spanish, English, or French, utilizing mobile applications for the management of adult individuals (over 18 years) with type 2 diabetes mellitus, and whose outcomes report the effects on glycated hemoglobin. The Cochrane Risk of Bias Tool will be used to assess the quality of the studies, and the Grading of Recommendations, Assessment, Development, and Evaluation (GRADE) methodology will be implemented to evaluate the certainty of the evidence.

**Results:**

The analysis will be conducted by observing the value of the glycated hemoglobin levels of the participants. Given that this data is a quantitative and continuous value, it facilitates the identification of the effects of the mobile applications used for the management of type 2 diabetes mellitus (T2DM) in adults. Furthermore, if sufficient data are available, a meta-analysis will be conducted using IBM-SPSS. The effect of the intervention will be estimated by the mean difference. All point estimates will be accompanied by 95% confidence intervals. A random effects model will be used. The heterogeneity of the results will be assessed using Cochrane’s Q and I2 statistics.

**Discussion:**

Considering that the quality of content and functionality of certain applications in the healthcare field is highly variable, it is necessary to evaluate the scientific evidence reported on the effect of the use of this type of technology in people with T2DM.

## Introduction

Non-communicable diseases (NCDs) are long-lasting and progress slowly, with heart diseases, strokes, cancer, respiratory diseases, and diabetes accounting for 60% of deaths worldwide [1–3]. However, when all NCDs are grouped together, it is impossible to determine the burden of each one individually. Although they are the most significant group in terms of preventable morbidity, disability, and mortality, according to the Pan American Health Organization, reducing mortality from these diseases requires generating various strategies, including modifying the structural conditions in the lives of those affected [2].

Considering the above, the low proportion of individuals with arterial hypertension and T2DM who manage to stabilize their levels following treatments for blood pressure control (<140/90) and glycated hemoglobin (HbA1c <7%) is concerning [4, 5]. It is observed that more than half of the people with these conditions do not achieve treatment goals, leading to increased complications, higher healthcare costs, diminished quality of life, and loss of human lives [4–6]. Several factors are identified as responsible for this issue, including limited training and/or education of individuals with chronic conditions in self-management [7], low adherence to treatment, barriers to accessing the healthcare system, and the lack of monitoring programs that involve comprehensive management of the illness in daily life (role management, dietary recommendations, physical exercise, smoking) [7, 8]. Additionally, there is a shortfall of human resources for ongoing follow-up and support of patients.

Therefore, the provision of healthcare services through remote telecommunications (eHealth) emerges as a hopeful and viable alternative to support these goals, bringing primary healthcare closer to the daily life activities of individuals with chronic conditions and their families. Among the eHealth solutions are mobile applications (Apps) for diabetes management, which are defined as "mobile phone software that accepts data (transmitted or manually entered) and provides feedback to patients on improved management (automated or by a healthcare professional)" [9], potentially offering new interventions to support self-management.

In recent years, there has been a surge in the number of apps, where mobile technology has impacted various aspects of daily life, changing the ways people communicate and work [10]. This presents an opportunity to integrate them into healthcare services as an extension of care, primarily for working individuals who find it difficult to attend health check-ups.

This integration could support behavioral changes that promote improvements in individuals’ health status, through the delivery of information, treatment plans, and goals that lead individuals to self-manage their health and illness [7, 11, 12]. However,the quality of content and functionality in available apps varies greatly, with many of them being unreliable [9]. Therefore, it is necessary to evaluate the scientific evidence reported on the effect of using this type of technology in people with T2DM.

## Materials and methods

### Objective and sample

The objective of the study is to describe the effect of mobile app usage on glycated hemoglobin for the management of adult patients with type 2 diabetes mellitus who participated in randomized controlled clinical trials.

To achieve this, a systematic review will be conducted on scientific publications from the last 5 years (January 2019 to October 2023), considering the following guiding question: How effective are mobile apps in managing people with type 2 diabetes mellitus? A descriptive and comparative analysis will be carried out on the differences in the level of HbA1c, looking for results in absolute values or changes between the beginning and the end of the intervention.

The systematic review protocol was designed following the recommendations of the Preferred Reporting Items for Systematic Reviews and Meta-Analyses protocols [13] and will adhere to the standard methodological norms established by the Cochrane Collaboration [14]. The corresponding protocol has been registered in the International Prospective Register of Systematic Reviews (PROSPERO) at the University of York. Its registration number is CRD42023475528.

### Search strategy

**Fig 1:**
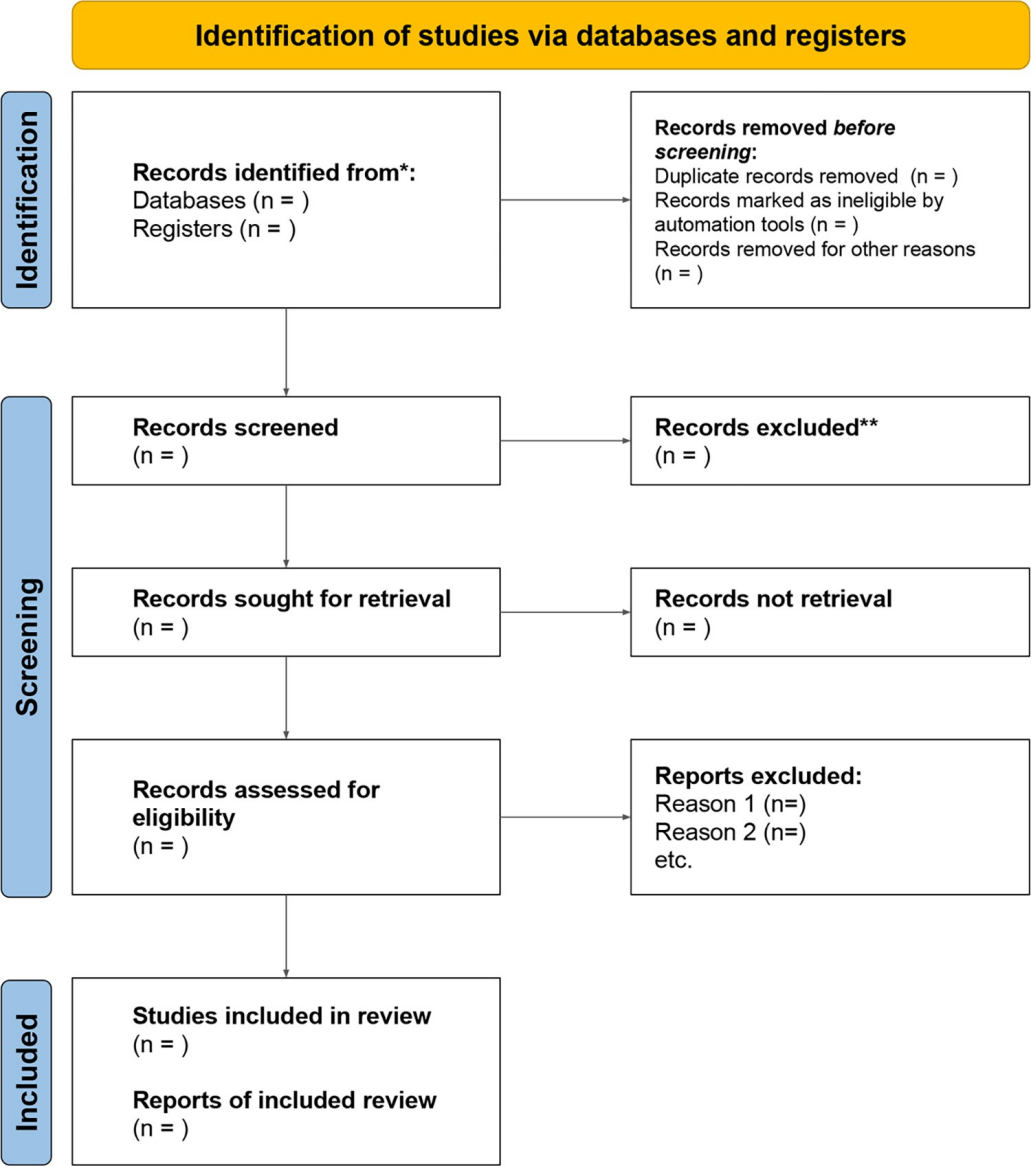
PRISMA flow diagram.

The search will be carried out in the MEDLINE (Ovid), Embase (Ovid), CINAHL (EBSCOhost), CENTRAL, WoS, Scopus, Epistemonikos and LILACS databases. The search strategy will be constructed with controlled and natural language. In addition, the Cochrane filter will be added to identify randomized controlled trials (RCTs). The bibliographic search will be conducted iteratively using keywords that correspond to the structure of the question, creating an adapted process by combining search terms using the Boolean operators "AND" to combine domains and "OR" for terms within the same domain. The search will be limited to the last 5 years. The results of the search strategy will be synthesized using a PRISMA flow diagram (Fig 1) [15].

A progressive approach will be used in the search for the inclusion of studies, which involves selecting the publications, first by analyzing the titles, and then evaluating the methodological aspects with an analysis of the abstracts. Two independent evaluators will review the selected texts. In cases where there is no agreement on a publication, a third author will be asked to act as a referee to decide on the selection (inclusion or exclusion of the study). The search results will be managed using Rayyan, an online platform that facilitates the process of detection of duplicates and assists in the blind assessment of eligibility among independent elimination reviewers [16, 17].

### Eligibility criteria

The review will include scientific articles reporting studies that present results from randomized controlled trials (RCTs), published in the last 5 years, with text in Spanish, English, or French, that utilize mobile applications for the management of adults (over 18 years old) with type 2 diabetes mellitus (T2DM), and whose results report effects on HbA1c. The inclusion of studies will not be limited when participants have coexisting chronic diseases. A mobile application is defined as software installed on the user’s cell phone, composed of modules in which various information is provided (use of medications, healthy habits, education about the disease, support for self-management, among other aspects).

On the other hand, publications that correspond to protocols, those not available in full text (for example, abstracts published in conference proceedings), and those that, referring to the use of technologies, use only text messages, emails, social networks, or phone calls, will be excluded from the study. Studies that do not provide data on the effects on HbA1c will also be excluded, as this is the primary outcome to be collected in this review, those whose design is not an RCT, and those that include a pediatric population among the participants.

### Assessment of the quality of included studies

#### Risk of bias assessment

The risk of bias assessment is considered an essential component in a systematic review [18]. The most used tool for assessing risk of bias in a systematic review of randomized clinical trials is the Cochrane Risk of Bias (RoB 2) tool [19]. In the present study, two reviewers will independently evaluate using the Cochrane RoB 2 tool. If discrepancies arise in any case, consensus will be used, and if it persists, a third reviewer will resolve the issue [20].

#### Certainty of evidence

The Grading of Recommendations, Assessment, Development and Evaluation (GRADE) methodology will be implemented to assess the certainty of the available evidence regarding the effect of using mobile applications on HbA1c for the control of adult patients with T2DM who participated in controlled clinical trials [21]. This procedure will also be carried out by two independent reviewers, with a third investigator joining as an arbitrator in cases where the two reviewers do not reach a consensus. The GRADE methodology allows for assigning a score to establish the certainty of the evidence. Factors that reduce the certainty include the perceived risk of bias, inconsistencies in the magnitude of the effect associated with heterogeneity, indirect estimation of the clinical effect, imprecision of the estimators, and the possibility of publication bias [21].

#### Data extraction

The information collected in this review will be grouped into two essential categories. One of them consists of the characteristics of the studies, where general aspects of the publications are described. The second contains the characteristics of the intervention. Therefore, the data that will be obtained from each study will include the following aspects, respectively:

#### Study characteristics

- First author’s surname.
- Year of publication.
- Language.
- Country or region where the study was conducted.

#### Characteristics of the intervention

- Study duration.
- Study design.
- Sample size.
- Participants age range.
- Objectives.
- Results.

### Analysis plan

The publications will be analyzed by observing the HbA1c level values of the participants in the RCTs. Since this data is quantitative and continuous, i.e., more objective, it facilitates the identification of the effects of mobile applications used for managing Type 2 Diabetes Mellitus in adults. This information will be transferred to an Excel spreadsheet. Furthermore, if sufficient data are available, a meta-analysis will be conducted using IBM-SPSS. The effect of the intervention will be estimated using the mean difference. The mean difference (MD) will be used as a summary statistic for continuous outcomes. The standardized mean difference (SMD) will be estimated if the results are reported on different scales. All point estimators will be accompanied by 95% confidence intervals (95% CI). A random-effects model will be used. Heterogeneity of results will be assessed using Cochrane’s Q and I2 statistics. The I2 statistic measures the between-study variation that cannot be attributed to chance and is expressed as a percentage. It is often classified as low, moderate, and high heterogeneity when the I2 value is <25%, 25 to 50%, and >50%, respectively. The fixed effects model will only be considered when the results are homogeneous (I2<25%) [22, 23].

Publication bias will be assessed using a funnel plot and Egger’s test. All analyses will be performed in Review Manager (RevMan) (version 5.2, Copenhagen: The Nordic Cochrane Centre, Cochrane Collaboration, 2012) [24]

### Study results

In this systematic review, the primary outcome will be the level of glycated hemoglobin (HbA1c) obtained after the intervention. The results reported in the studies, whether they are absolute values or changes in levels between the start and end of the intervention, will be considered.

## Discussion

There is a variety of mHealth tools aimed at managing health remotely to enhance promotion, prevention, diagnosis, treatment, and disease monitoring. However, there is limited evidence of effectiveness for glycemic control with the use of mobile applications focused on remote diabetes care [25]. Additionally, it has been described that the use of technologies can be useful for improvements in HbA1c levels and the reduction of hypoglycemia events [26]. In this sense, randomized clinical trials are shown as a rigorous process for studying the effects of a particular intervention. Thus, having information from a systematic review of the use of mobile applications aimed at controlling type 2 diabetes using such investigative design can be a contribution to determining the limitations and successes obtained with this technology.

## Supporting information

S1 Checklist (PDF)
