# Supplementary material for: How effective are mobile apps in managing people with type 2 diabetes mellitus? A systematic literature review protocol
Source: PLoS One. 2024 Apr 25;19(4):e0301523. doi: 10.1371/journal.pone.0301523 (PMC11045108; doi:10.1371/journal.pone.0301523)
Supplement: S1 Checklist — (PDF) [file pone.0301523.s001.pdf]

ESTRATEGIA DE BÚSQUEDA

OVID Medline Epub Ahead of Print, In-Process & Other Non-Indexed Citations, Ovid MEDLINE(R) Daily and Ovid MEDLINE(R) 1946 to Present

1 exp Diabetes Mellitus, Type 2/
2 (MODY or NIDDM or T2DM or T2D).tw.
3 (non insulin$ depend$ or noninsulin$ depend$ or noninsulin?depend$ or non insulin?depend$).tw.
4 ((typ? 2 or typ? II or typ?2 or typ?II) adj3 diabet$).tw.
5 (((late or adult$ or matur$ or slow or stabl$) adj3 onset) and diabet$).tw.
6 or/1-5
7 Text Messaging/
8 ((mms or sms) and (text$ or messag$)).tw.
9 (multimedia messag$ service$ or short messag$ service$).tw.
10 (text messag$ or texting).tw.
11 exp Cellular Phone/
12 ((car or cell$ or smart or mobile) adj3 phone$).tw.
13 (carphone$ or cellphone$ or smartphone$ or mobilephone$).tw.
14 (iphone$ or ipod$ or podcast$ or ipad$ or android$ or blackberr$ or palm pilot$).tw.
15 exp Computers, Handheld/
16 (pda$ or personal digital assistant$).tw.
17 (tablet adj6 (computer or pc)).tw.
18 ((wireless or handheld) adj3 (device$ or technolog$)).tw.
19 Telemedicine/
20 (telemedicine or tele-medicine or telehealth or tele-health or telemonitor$ or tele-monitor$ or ehealth or e-health or mhealth or m-health).tw.
21 (mobile adj3 health$).tw.
22 ((computer or online or internet or web) adj3 (learn$ or educat$ or instruct$)).tw.
23 Electronic Mail/
24 exp Internet/
25 (web or website$ or internet).tw.
26 (social adj3 (media or network$)).tw.
27 exp Mobile Applications/
28 (mobile adj2 app$).tw.
29 or/7-
30 6 and 29
31 randomized controlled trial.pt.
32 controlled clinical trial.pt.
33 randomized.ab.
34 placebo.ab.
35 clinical trials as topic.sh.
36 randomly.ab.
37 trial.ti.
38 or/31-37
39 exp animals/ not humans.sh.
40 38 not 39
41 30 and 40
42 41 and 2019:2023.(sa_year).

Embase <1974 to 2023 October 25>

1 exp non insulin dependent diabetes mellitus/
2 (MODY or NIDDM or T2DM or T2D).tw.

3 (non insulin$ depend$ or noninsulin$ depend$ or noninsulin?depend$ or non insulin?depend$).tw.
4 ((typ? 2 or typ? II or typ?2 or typ?II) adj3 diabet$).tw.
5 (((late or adult$ or matur$ or slow or stabl$) adj3 onset) and diabet$).tw.
6 or/1-5
7 text messaging/
8 ((mms or sms) and (text$ or messag$)).tw.
9 (multimedia messag$ service$ or short messag$ service$).tw.
10 (text messag$ or texting).tw.
11 exp mobile phone/
12 ((car or cell$ or smart or mobile) adj3 phone$).tw.
13 (carphone$ or cellphone$ or smartphone$ or mobilephone$).tw.
14 (iphone$ or ipod$ or podcast$ or ipad$ or android$ or blackberr$ or palm pilot$).tw.
15 exp personal digital assistant/
16 (pda$ or personal digital assistant$).tw.
17 (tablet adj6 (computer or pc)).tw.
18 ((wireless or handheld) adj3 (device$ or technolog$)).tw.
19 telemedicine/
20 (telemedicine or tele-medicine or telehealth or tele-health or telemonitor$ or tele-monitor$ or ehealth or e-health or mhealth or m-health).tw.
21 (mobile adj3 health$).tw.
22 ((computer or online or internet or web) adj3 (learn$ or educat$ or instruct$)).tw.
23 e-mail/
24 exp Internet/
25 (web or website$ or internet).tw.
26 (social adj3 (media or network$)).tw.
27 exp mobile application/
28 (mobile adj2 app$).tw.
29 or/7-
30 6 and 29
31 exp randomized controlled trial/
32 controlled clinical trial/
33 random$.ti,ab.
34 randomization/
35 intermethod comparison/
36 placebo.ti,ab.
37 (compare or compared or comparison).ti,ab.
38 ((evaluated or evaluate or evaluating or assessed or assess) and (compare or compared or comparing or comparison)).ab.
39 (open adj label).ti,ab.
40 ((double or single or doubly or singly) adj (blind or blinded or blindly)).ti,ab.
41 double blind procedure/
42 parallel group$1.ti,ab.
43 (crossover or cross over).ti,ab.
44 ((assign$ or match or matched or allocation) adj5 (alternate or group$1 or intervention$1 or patient$1 or subject$1 or participant$1)).ti,ab.
45 (assigned or allocated).ti,ab.
46 (controlled adj7 (study or design or trial)).ti,ab.
47 (volunteer or volunteers).ti,ab.
48 human experiment/
49 trial.ti.
50 or/31-49
51 (random$ adj sampl$ adj7 ("cross section$" or questionnaire$1 or survey$ or database$1)).ti,ab. not (comparative study/ or controlled study/ or randomi?ed controlled.ti,ab. or randomly assigned.ti,ab.)

52 cross-sectional study/ not (exp randomized controlled trial/ or controlled clinical trial/ or controlled study/ or randomi?ed controlled.ti,ab. or control group$1.ti,ab.)
53 (((case adj control$) and random$) not randomi?ed controlled).ti,ab.
54 systematic review.ti,ab. not (trial or study).ti.
55 (nonrandom$ not random$).ti,ab.
56 "random field$".ti,ab.
57 (review.ab. and review.pt.) not trial.ti.
58 "we searched".ab. and (review.ti. or review.pt.)
59 "update review".ab.
60 (databases adj4 searched).ab.
61 (rat or rats or mouse or mice or swine or porcine or murine or sheep or lambs or pigs or piglets or rabbit or rabbits or cat or cats or dog or dogs or cattle or bovine or monkey or monkeys or trout or marmoset$1).ti. and animal experiment/
62 animal experiment/ not (human experiment/ or human/)
63 or/51-62
64 50 not 63
65 30 and 64
66 65 and 2019:2023.(sa_year).

CINAHL (EBSCOhost)

S1 (MH "Diabetes Mellitus, Type 2")
S2 TX MODY or NIDDM or T2DM or T2D
S3 TX (non insulin* depend* or noninsulin* depend* or noninsulin#depend* or non insulin#depend*)
S4 TX ((typ# 2 or typ# II or typ#2 or typ#II) N3 diabet*)
S5 TX (((late or adult* or matur* or slow or stabl*) N3 onset) and diabet*)
S6 S1 OR S2 OR S3 OR S4 OR S5
S7 (MH "Text Messaging")
S8 TX ((mms or sms) and (text* or messag*))
S9 TX (multimedia messag* service* or short messag* service*)
S10 TX (text messag* or texting)
S11 (MH "Cellular Phone+")
S12 TX ((car or cell* or smart or mobile) N3 phone*)
S13 TX (carphone* or cellphone* or smartphone* or mobilephone*)
S14 TX (iphone* or ipod* or podcast* or ipad* or android* or blackberr* or palm pilot*)
S15 (MH "Computers, Hand-Held+")
S16 TX (pda* or personal digital assistant*)
S17 TX (tablet N6 (computer or pc))
S18 TX ((wireless or handheld) N3 (device* or technolog*))
S19 (MH "Telemedicine")
S20 TX (telemedicine or tele-medicine or telehealth or tele-health or telemonitor* or tele-monitor* or ehealth or e-health or mhealth or m-health)
S21 TX (mobile N3 health*)
S22 TX ((computer or online or internet or web) N3 (learn* or educat* or instruct*))
S23 (MH "Email")
S24 (MH "Internet+")
S25 TX (web or website* or internet)
S26 TX (social N3 (media or network*))
S27 (MH "Mobile Applications")
S28 TX (mobile N2 app*)
S29 S7 OR S8 OR S9 OR S10 OR S11 OR S12 OR S13 OR S14 OR S15 OR S16 OR S17 OR S18 OR S19 OR S20 OR S21 OR S22 OR S23 OR S24 OR S25 OR S26 OR S27 OR S28
S30 S6 AND S29

S31 ((MH "Experimental Studies+") OR (MH "Multicenter Studies") OR (MH "Random Sample+") OR (MH "Placebos") OR (MH "Control (Research)+") OR (MH "Crossover Design") OR ((TI random* OR AB random*) OR (TI sham OR AB sham) OR (TI placebo* OR AB placebo*)) OR (((TI singl* OR AB singl*) OR (TI doubl* OR AB doubl*)) W1 ((TI blind* OR AB blind*) OR (TI dumm* OR AB dumm*) OR (TI mask* OR AB mask*))) OR (((TI tripl* OR AB tripl*) OR (TI trebl* OR AB trebl*)) W1 ((TI blind* OR AB blind*) OR (TI dumm* OR AB dumm*) OR (TI mask* OR AB mask*))) OR ((TI control* OR AB control*) N3 ((TI study OR AB study) OR (TI studies OR AB studies) OR (TI trial* OR AB trial*) OR (TI group* OR AB group*))) OR ((TI clinical OR AB clinical) N3 ((TI study OR AB study) OR (TI studies OR AB studies) OR (TI trial* OR AB trial*))) OR ((TI Nonrandom* OR AB Nonrandom*) OR (TI "non random*" OR AB "non random*") OR (TI "non-random*" OR AB "non-random*") OR (TI "quasi-random*" OR AB "quasi-random*") OR (TI quasirandom* OR AB quasirandom*)) OR ((TI phase OR AB phase) N3 ((TI study OR AB study) OR (TI studies OR AB studies) OR (TI trial* OR AB trial*))) OR (((TI crossover OR AB crossover) OR (TI "cross-over" OR AB "cross-over")) N3 ((TI study OR AB study) OR (TI studies OR AB studies) OR (TI trial* OR AB trial*))) OR (((TI multicent* OR AB multicent*) OR (TI "multi-cent*" OR AB "multi-cent*")) N3 ((TI study OR AB study) OR (TI studies OR AB studies) OR (TI trial* OR AB trial*))) OR (TI allocated OR AB allocated) OR (((TI "open label" OR AB "open label") OR (TI "open-label" OR AB "open-label")) N5 ((TI study OR AB study) OR (TI studies OR AB studies) OR (TI trial* OR AB trial*))) OR (((TI equivalence OR AB equivalence) OR (TI superiority OR AB superiority) OR (TI "non-inferiority" OR AB "non-inferiority") OR (TI noninferiority OR AB noninferiority)) N3 ((TI study OR AB study) OR (TI studies OR AB studies) OR (TI trial* OR AB trial*))) OR ((TI "pragmatic study" OR AB "pragmatic study") OR (TI "pragmatic studies" OR AB "pragmatic studies")) OR (((TI pragmatic OR AB pragmatic) OR (TI practical OR AB practical)) N3 (TI trial* OR AB trial*)) OR (((TI quasiexperimental OR AB quasiexperimental) OR (TI "quasi-experimental" OR AB "quasi-experimental")) N3 ((TI study OR AB study) OR (TI studies OR AB studies) OR (TI trial* OR AB trial*))) OR (TI trial))
S32 S30 AND S31
S33 S30 AND S31 Limiters - Published Date: 20190101-20231231

CENTRAL (Cochrane Library)

#1 MeSH descriptor: [Diabetes Mellitus, Type 2] explode all trees
#2 (MODY or NIDDM or T2DM or T2D)
#3 (non insulin* depend* or noninsulin* depend* or noninsulin?depend* or non insulin?depend)
#4 ((typ? 2 or typ? II or typ?2 or typ?II) near/3 diabet*)
#5 (((late or adult* or matur* or slow or stabl*) near/3 onset) and diabet*)
#6 {OR #1-#5}
#7 MeSH descriptor: [Text Messaging] explode all trees
#8 ((mms or sms) and (text* or messag*))
#9 (multimedia messag* service* or short messag* service*)
#10 (text messag* or texting)
#11 MeSH descriptor: [Cell Phone] explode all trees
#12 ((car or cell* or smart or mobile) near/3 phone*)
#13 (carphone* or cellphone* or smartphone* or mobilephone*)
#14 (iphone* or ipod* or podcast* or ipad* or android* or blackberr* or palm pilot*)
#15 MeSH descriptor: [Computers, Handheld] explode all trees
#16 (pda* or personal digital assistant*)
#17 (tablet near/6 (computer or pc))
#18 ((wireless or handheld) near/3 (device* or technolog*))
#19 MeSH descriptor: [Telemedicine] explode all trees
#20 (telemedicine or tele-medicine or telehealth or tele-health or telemonitor* or tele-monitor* or ehealth or e-health or mhealth or m-health)
#21 (mobile near/3 health*)
#22 ((computer or online or internet or web) near/3 (learn* or educat* or instruct*))
#23 MeSH descriptor: [Electronic Mail] explode all trees
#24 MeSH descriptor: [Internet] explode all trees
#25 (web or website* or internet)
#26 (social near/3 (media or network*))
#27 MeSH descriptor: [Mobile Applications] explode all trees
#28 (mobile near/2 app*)

#29 {OR #7-#28}
#30 #6 AND #29
#31 Trials
#32 2019-2023

SCOPUS

(((TITLE-ABS-KEY ("type 2 diabetes mellitus") OR TITLE-ABS-KEY (mody OR niddm OR t2dm OR t2d) OR TITLE-ABS-KEY ("non insulin* depend*" OR "noninsulin* depend*" OR "noninsulin*depend*" OR "non insulin*depend*"))) AND ((TITLE-ABS-KEY ("text messaging") OR TITLE-ABS-KEY ((mms OR sms) AND (text* OR messag*)) OR TITLE-ABS-KEY ("multimedia messag* service*" OR "short messag* service*") OR TITLE-ABS-KEY ("text messag*" OR texting) OR TITLE-ABS-KEY ("cellular phone") OR TITLE-ABS-KEY (carphone* OR cellphone* OR smartphone* OR mobilephone*) OR TITLE-ABS-KEY (iphone* OR ipod* OR podcast* OR ipad* OR android* OR blackberr* OR "palm pilot*") OR TITLE-ABS-KEY (pda* OR "personal digital assistant*") OR TITLE-ABS-KEY (telemedicine OR "tele-medicine" OR telehealth OR "tele-health" OR telemonitor* OR "tele-monitor*" OR ehealth OR "e-health" OR "mhealth" OR "m-health") OR TITLE-ABS-KEY ("electronic mail" OR "e-mail" OR email) OR TITLE-ABS-KEY (web OR website* OR internet) OR TITLE-ABS-KEY ("mobile app*")))) AND (TITLE-ABS-KEY (random* OR sham OR placebo*) OR TITLE-ABS-KEY ((singl* OR doubl*) W/1 (blind* OR dumm* OR mask*)) OR TITLE-ABS-KEY ((tripl* OR trebl*) W/1 (blind* OR dumm* OR mask*)) OR TITLE-ABS-KEY (control* W/3 (study OR studies OR trial* OR group*)) OR TITLE-ABS-KEY (clinical W/3 (study OR studies OR trial*)) OR TITLE-ABS-KEY (nonrandom* OR "non random*" OR non-random* OR quasi-random* OR quasirandom*) OR TITLE-ABS-KEY (phase W/3 (study OR studies OR trial*)) OR TITLE-ABS-KEY ((crossover OR cross-over) W/3 (study OR studies OR trial*)) OR TITLE-ABS-KEY ((multicent* OR multi-cent*) W/3 (study OR studies OR trial*)) OR TITLE-ABS (allocated) OR TITLE-ABS-KEY (("open label" OR open-label) W/5 (study OR studies OR trial*)) OR TITLE-ABS-KEY ((equivalence OR superiority OR non-inferiority OR noninferiority) W/3 (study OR studies OR trial*)) OR TITLE-ABS-KEY ("pragmatic study" OR "pragmatic studies") OR TITLE-ABS-KEY ((pragmatic OR practical) W/3 trial*) OR TITLE-ABS-KEY ((quasiexperimental OR quasi-experimental) W/3 (study OR studies OR trial*)) OR TITLE (trial) OR KEY (trial)) AND PUBYEAR > 2018 AND PUBYEAR < 2024

WoS

#1 "type 2 diabetes mellitus" (Topic) or mody OR niddm OR t2dm OR t2d (Topic) or "non insulin* depend*" OR "noninsulin* depend*" OR "noninsulin*depend*" OR "non insulin*depend*" (Topic)
#2 "text messaging" (Topic) or (mms OR sms) AND (text* OR messag*) (Topic) or "multimedia messag* service*" OR "short messag* service*" (Topic) or "text messag*" OR texting (Topic) or "cellular phone" (Topic) or carphone* OR cellphone* OR smartphone* OR mobilephone* (Topic) or iphone* OR ipod* OR podcast* OR ipad* OR android* OR blackberr* OR "palm pilot*" (Topic) or pda* OR "personal digital assistant*" (Topic) or telemedicine OR "tele-medicine" OR telehealth OR "tele-health" OR telemonitor* OR "tele-monitor*" OR ehealth OR "e-health" OR "mhealth" OR "m-health" (Topic) or "electronic mail" OR "e-mail" OR email (Topic) or web OR website* OR internet (Topic) or "mobile app*" (Topic)
#3 #1 AND #2
#4 TS=((random* or control* or trial or compar* or group or groups or therapy or treatment or intervention))
#5 #3 AND #4
#6 #3 AND #4 and 2019 or 2020 or 2021 or 2022 or 2023 (Publication Years)

Epistemonikos

(advanced_title_en:("type 2 diabetes mellitus" OR mody OR niddm OR t2dm OR t2d OR "non insulin* depend*" OR "noninsulin* depend*" OR "noninsulin*depend*" OR "non insulin*depend*") OR advanced_abstract_en:("type 2 diabetes mellitus" OR mody OR niddm OR t2dm OR t2d OR "non insulin* depend*" OR "noninsulin* depend*" OR "noninsulin*depend*" OR "non insulin*depend*")) AND (advanced_title_en:("text messaging" OR ((mms OR sms) AND (text* OR messag*)) OR "multimedia messag* service*" OR "short messag* service*" OR "text messag*" OR texting OR "cellular phone" OR carphone* OR cellphone* OR smartphone* OR mobilephone* OR iphone* OR ipod* OR podcast* OR ipad* OR android* OR blackberr* OR "palm pilot*" OR pda* OR "personal digital assistant*" OR telemedicine OR "tele-medicine" OR telehealth OR "tele-health" OR telemonitor* OR "tele-monitor*" OR ehealth OR "e-health" OR

"mhealth" OR "m-health" OR "electronic mail" OR "e-mail" OR email OR web OR website* OR internet OR "mobile app*") OR advanced_abstract_en:("text messaging" OR ((mms OR sms) AND (text* OR messag*)) OR "multimedia messag* service*" OR "short messag* service*" OR "text messag*" OR texting OR "cellular phone" OR carphone* OR cellphone* OR smartphone* OR mobilephone* OR iphone* OR ipod* OR podcast* OR ipad* OR android* OR blackberr* OR "palm pilot*" OR pda* OR "personal digital assistant*" OR telemedicine OR "tele-medicine" OR telehealth OR "tele-health" OR telemonitor* OR "tele-monitor*" OR ehealth OR "e-health" OR "mhealth" OR "m-health" OR "electronic mail" OR "e-mail" OR email OR web OR website* OR internet OR "mobile app*")) [Filters: classification=primary-study, protocol=no, min_year=2019, max_year=2023]

LILACS

(("type 2 diabetes mellitus") OR (or mody OR niddm OR t2dm OR t2d)) AND (("mobile app*")) AND (type_of_study:("clinical_trials")) AND (year_cluster:[2019 TO 2023])
